# Decadence of the State Medical Association:—Why?

**Published:** 1898-06

**Authors:** 


### Decadence of the State Medical Association:—Why?

Paganini is said to have played all tunes on one string; but—he made harmony. The Texas State Medical Association are still sawing away on the one string—regulating the practice—and are producing discord.

The Texas State Medical Association, which a few years ago carried six hundred or eight hundred active members on its roll, and paid for 750 volumes of a cloth bound edition of transactions of 792 pages $1185 without "busting" the treasury, now numbers, by actual count, 243 members, according to the transactions for 1897, now before us. To this must be added, however, sixty recruits who enlisted at the recent Houston meeting, many of them being old members, dropped from the roll, making a grand total, after *thirty years*, of 303.

This is about six per cent. of the medical profession of Texas, according to the best estimate that can be made. (See Polk's Directory.)

There surely must be a reason for this want of cohesion, or want of popularity,—and it behoves the friends of the Association to look into it. The same conditions exist as formerly, except that the cost of membership is less. (There is "no way to enforce collections," the secretary complains; and never was, we will state parenthetically.) Is it mismanagement? Is it inherent defect in the laws of the Association? or is an explanation of the "decline and fall off" to be found in the persistent efforts of those who seem to be in charge to hand over to the homeopaths and eclectics the entire medical profession of the State, ninety-four per cent. of whom do not belong to this body? to "homologate" with that element of quacks; to recognize them, and take them to their loving embrace as physicians and equals? I believe that this is the secret of the evident unpopularity of the Association, as evidenced by the steady decline in membership. As fast as recruits are enlisted, an equal number, or a greater number, is dropped; so that at the

*thirtieth* annual session the secretary called the roll of 243, to which perhaps one hundred responded!

In former articles in which it was sought to show the folly—the humiliation—the degredation of medicine and the contravention of the spirit and letter of the Code, if the State Association should recognize homeopathy as a "school of medicine," and homeopaths as physicians, those who "practice an exclusive dogma," the writer claimed that the State Medical Association is not, in any sense, a representative body, and has no right to commit the profession of the State to any such policy. However, there is little room to fear that the legislature will grant these gentlemen what they have so often refused—the right to regulate anybody's practice except their own. The JOURNAL has reiterated this—the position of past legislatures—until it has become monotonous.

But the Association, it seems, will take no refusal. They, undismayed by the ill favor with which all previous attempts have been received; not discouraged by repeated refusals, are up and at it again, with the same old tune: a State board or council of medical examiners, to be composed of the Attorney General, the State Health Officer, the president of the University Regents and the presidents respectively of the regulars, the irregulars and the defectives; that is to say, the presidents of the associations of the respective "schools of medicine"(?) (The Code knows no "school" and recognizes no "pathy"); or, to be more specific, the president of the Texas State Medical Association (303 members), the president of the Homeopathic Medical (?) Association (numbering twenty-seven strong last meeting), and the president of the Eclectic Medical Association, whatever that may be.

In support of the assertion that the State Medical Association is not a representative body of the profession of the State, I cite here from the report of the committee on organization the following; (See transactions 1897, page 29):

"Of 246 counties in the State, 91, or 37 per cent., are represented in the membership of the State Medical Association."

As showing the distribution of this membership, we will state, (vide report of committee on organization, loc. cit.) that 108 members, or over one-third of the entire membership, live in extreme North Texas, in twenty-six counties in latitude 32 to 34, longitude 95 to 99 (and both the president-elect and the late president are from North Texas); while in East Texas, latitude

30 to 32, longitude 93½ to 96, in twenty-three counties, there are eighteen members.

In Southwest Texas, latitude 27 to 30, longitude 99 to 101½, in fourteen counties, there is one member or representative in the Association.

In this mending of their hold; this rolling up of their sleeves and spitting on their hands, so to speak, by the Association in their fifteenth or twentieth petition to the legislature and their determination to force the "regulating" business, they remind us of the pigeon that, having had his perceptive faculties destroyed by the removal of part of the brain, walked around and around in a ring all day, stumbling over the same block in its path every time it came to it.

The performance of the State Association has grown monotonous. They make the same mistake every time, the stumbling block being the repeated refusal of the legislature to accord to this body the power to dictate to the medical profession the terms on which they shall practice in Texas. Why can they not let the homeopaths alone, and seek to amend the existing law so as to make it effective; or to enforce it, defective as it is? Homeopaths—those who "trade on the name" and "practice exclusive dogma"—are not physicians, and have nothing in common with physicians. Homeopathy is no part of medicine, and yet the association of regular physicians, it seems, rather than not "regulate the practice," seek to form an alliance with this, a very large element of quacks, to suppress other quacks.

Well, it is no funeral of mine; I did all I could to defeat the other bill while it was pending before the convention,—fought it single handed—because I believed that the Association humbled and compromised itself in the step it proposed to take. I shall cease to oppose anything that body proposes to do, but I do think the great body of the profession should be heard, and should not have their hands tied in any such manner as is proposed by the valliant and resolute 303.

